# Bridging the divide: barriers and facilitators to equitable community-academic partnerships in health research

**DOI:** 10.3389/fpubh.2025.1617908

**Published:** 2025-09-16

**Authors:** Maissa Khatib, Rushabh Shah, Surbhi Mendhe, Corrie Whisner, Matthew Buman

**Affiliations:** College of Health Solutions, Arizona State University, Phoenix, AZ, United States

**Keywords:** community-based participatory research, community-academic partnerships, qualitative research, health equity, health research

## Abstract

**Objective:**

This study explores best practices for fostering sustainable and equitable community-academic partnerships in public health, focusing on identifying key barriers, facilitators, and health priorities among historically underrepresented populations in Arizona—including Latinx, Native American, African American, LGBTQ+, immigrant, and refugee communities. Grounded in the Community-Based Participatory Research (CBPR) model, the study emphasizes equity, shared power, and collaborative engagement.

**Method:**

Using a descriptive qualitative approach, the study draws on 21 in-depth interviews—combining semi-structured and vignette-based formats—with academic researchers, community members, and community partners. Thematic analysis was conducted across three CBPR-aligned domains: (1) challenges to equitable partnerships, (2) practices that foster collaboration, and (3) community-identified health priorities.

**Results:**

Findings revealed seven key themes across the three domains. Under Domain 1 (Challenges to Equitable Partnerships), participants identified three primary barriers: (1) misaligned funding structures and short-term grants, (2) institutional bureaucracy and academic incentive systems that deprioritize community engagement, and (3) mismatched timelines and priorities between academic researchers and community partners. Domain 2 (Practices that Foster Collaboration) highlighted three themes that support effective partnerships: (4) trust-building through cultural humility and transparency, (5) shared decision-making and mutual empowerment, and (6) commitment to sustained engagement beyond project timelines. Finally, Domain 3 (Community-Identified Health Priorities) surfaced a seventh theme: (7) mental health stigma, lack of culturally responsive care, and structural barriers such as poverty, immigration status, and geographic isolation.

**Conclusion:**

This study contributes to the literature on equitable community-academic partnerships by offering actionable strategies grounded in lived experience. Emphasizing continuous engagement, co-leadership, and alignment with community-defined priorities, these findings support the development of culturally relevant, context-specific interventions that address health disparities in historically marginalized populations. The lessons from Arizona are transferable to other underserved regions, reinforcing the need for structural reforms and relationship-centered research practices.

## 1 Introduction

Addressing today’s most pressing health challenges requires a comprehensive understanding of the complex interplay between biological, behavioral, genetic, environmental, and sociocultural factors. These determinants do not act in isolation; their cumulative effects are particularly pronounced among historically marginalized and underserved populations. Communities such as Latinx, Native American, African American, LGBTQ+, low-income, immigrant, and refugee groups often face systemic barriers to healthcare access and experience disproportionately high rates of chronic illness and adverse health outcomes (1, 2).

Traditional research approaches—often top-down in design—have failed to adequately address these disparities. By excluding the voices of affected communities, such models frequently produce interventions that lack cultural relevance, sustainability, and community buy-in (3, 4). In contrast, community-engaged approaches, such as Community-Based Participatory Research (CBPR) and Community-Engaged Research (CEnR), have gained recognition for their potential to bridge research and practice, improve relevance, and empower communities to drive health equity (5, 6).

The Centers for Disease Control and Prevention (CDC) defines community engagement as “the process of working collaboratively with groups of people who are affiliated by geographic proximity, special interests, or similar situations with respect to issues affecting their well-being.” This process spans a continuum from outreach and consultation to collaboration and shared leadership. Active participation by communities—particularly in identifying priorities and shaping interventions—can produce more equitable, impactful outcomes and help build long-term trust.

Academic-community partnerships are increasingly recognized as essential for advancing health equity, especially in addressing persistent disparities among underserved populations. These collaborations aim to bridge the gap between research and practice by integrating community knowledge and priorities (7). However, their success depends on early, intentional strategies and strong relational foundations (8). While shared goals and aligned values often motivate participation, such partnerships must also navigate institutional power dynamics (9, 10). Trust, transparency, and the cultivation of “good solid relationships” have emerged as critical to long-term collaboration and program success (11). Notably, researchers and community members often bring distinct yet complementary motivations and concerns, underscoring the need for mutual understanding and responsive, equity-centered engagement strategies (12).

Despite the promise of community-engaged approaches, persistent challenges hinder their effective implementation. These include historical mistrust, power imbalances, inadequate researcher preparation, and lack of compensation for community partners (13, 14). Sustaining equitable partnerships requires intentional investment in reciprocity, cultural humility, and shared ownership of both the process and its outcomes.

The impetus for this project stemmed from the unique sociopolitical and demographic context of Arizona—a state marked by deep geographic and socioeconomic health disparities. Arizona presents a particularly compelling context for examining community engagement in public health. As one of the most demographically diverse and fastest-growing states in the U. S., Arizona is home to 7.5 million residents, including significant Latinx, Native American, African American, LGBTQ+, immigrant, and refugee populations. Many of these communities remain underrepresented in health research due to persistent linguistic, cultural, geographic, and socioeconomic barriers (15, 16). The stark health disparities in the state—for example, the well-documented life expectancy gap between affluent North Scottsdale and underserved South Phoenix—highlight the urgency of addressing systemic inequities in healthcare access and outcomes.

In response to these disparities, Arizona State University (ASU) has launched several initiatives to promote health equity through community-engaged research. These include collaborations with Maricopa County to address geographic health disparities, efforts to reduce COVID-19-related inequities among American Indian, Latinx, and African American communities, and participation in the AZ Healthy Tomorrow initiative to expand healthcare access and strengthen the public health workforce. These efforts, alongside ASU’s College of Health Solutions’ institutional commitment to centering community voices in research, provided the impetus for this study.

While national literature has documented common barriers to research participation—including mistrust of academic institutions, logistical challenges such as childcare and transportation, limited work flexibility, and language or cultural mismatches (17–19)— there is limited understanding of how these factors manifest within Arizona’s distinct social and geographic landscape. Given the state’s unique demographic profile, such barriers may present differently across Latinx, Native American, African American, LGBTQ+, immigrant, and refugee communities. For instance, although Native American and Latinx populations in Arizona disproportionately experience chronic conditions such as diabetes and cardiovascular disease, they remain largely excluded from clinical research—limiting the relevance and effectiveness of public health interventions (20, 21). As Burns et al. (22) argue, public participation and engagement are critical for translating research into meaningful, measurable outcomes.

This study seeks to generate insights from community members, community partners, and academic researchers regarding the local barriers to meaningful community engagement in health research, as well as opportunities to strengthen community-academic partnerships.

Guided by the Community-Based Participatory Research (CBPR) conceptual model (6, 23), which emphasizes equitable partnerships, co-learning, shared power, and mutual benefit — this study explores strategies for building and sustaining community-academic partnerships that elevate the voices of historically underrepresented populations in Arizona. Drawing on qualitative data from in-depth interviews, it identifies key barriers, facilitators, and best practices in initiating and maintaining these partnerships within the state’s unique sociocultural and geographic context.

## 2 Methodology

### 2.1 Study design and research question

This descriptive qualitative study explores best practices for equitable community engagement by drawing on data from 21 in-depth interviews with academic researchers and community interest holders and community members. Across Arizona. The central research question guiding this inquiry is: What are the key barriers and facilitators to initiating and sustaining equitable community-academic partnerships in health research among historically underrepresented populations in Arizona? This question aims to illuminate the components of effective, community-responsive partnerships and identify the factors that enable or hinder long-term, equitable collaboration. A descriptive qualitative design was chosen to understand the nuanced experiences and perceptions of both researchers and community members regarding collaboration. This design is well-suited for exploring real-world phenomena from participants’ perspectives and for capturing the depth of experience without overinterpretation (24).

### 2.2 Sampling and participants

Following approval from the Arizona State University (ASU) Institutional Review Board (IRB), participants were recruited through purposive sampling from both ASU’s College of Health Solutions and surrounding community networks. Recruitment strategies included targeted emails distributed via institutional listservs, announcements posted on Slack channels, and public outreach through the College’s official Facebook page. In alignment with the study’s aim to explore complex, context-specific experiences, the target sample size was 20–30 participants. This range is consistent with qualitative research best practices, which prioritize depth of insight over statistical generalizability (25).

Eligibility criteria required participants to be 18 years of age or older and to identify as either (1) a community member or community partners engaged in health-related work or lived experience, or (2) an academic health researcher affiliated with ASU. The final sample included 21 participants: 9 academic researchers and 12 community members (see Table 1). Among the researchers, six had prior experience conducting community-engaged research, while two had such experience. These two researchers were included to capture a range of perspectives on institutional readiness, perceived barriers to engagement, and gaps in training or support. Their insights help illuminate structural and cultural challenges within academic settings that may hinder the development of equitable academic-community partnerships. The 12 community participants were drawn from diverse neighborhoods across the Phoenix metropolitan area. This group comprised six individuals deeply involved in grassroots community efforts and six community partners working across a range of sectors, including nutrition, healthcare, education, resettlement services, sports, and pharmaceuticals. The diversity of roles and experiences represented in the sample allowed for a wide spectrum of perspectives on community engagement in health research, including insights into both barriers and strategies for successful partnerships.

**Table 1:** Characteristics of study participants (*N* = 21).

Category	*n*	Description
Gender		
Female	15	–
Male	6	–
Participant role		
ASU-affiliated researchers	8	Faculty and research staff at ASU
Community members	13	Residents and stakeholders from Phoenix metropolitan area
Researcher experience (*n* = 8)		
No prior experience in community-engaged research	2	–
Prior experience in community-based research	6	–
Senior administrative role (project manager)	1	Oversees funded health research initiatives
Community member background (*n* = 13)		
Actively engaged community members	6	Residents active in community activities
Community leaders/stakeholders	6	Leaders in nutrition, education, healthcare, sports, pharmaceuticals, resettlement, integration

### 2.3 Data collection procedures

Data collection for this study involved two rounds of interviews, conducted using a combination of semi-structured protocols and vignette-based techniques, complemented by demographic questionnaires and observational field notes. These methods were designed to generate rich, context-specific insights while ensuring participant comfort and confidentiality.

### 2.4 First round – semi-structured interviews

The initial interview guide was developed through a review of the literature on community-academic partnerships, with particular attention to well-documented challenges and enablers such as trust-building, power imbalances, representation, and sustainability. Insights from this evidence base informed the selection of key domains and the formulation of guiding questions for the first round of interviews. The initial round of data collection involved semi-structured interviews guided by an open-ended protocol. This format allowed participants—both academic researchers and community members—to reflect on their experiences with community-academic partnerships and to articulate their priorities related to health and engagement. The semi-structured approach balanced consistency across interviews with the flexibility to follow participant-driven narratives and emerging topics of interest (26).

### 2.5 Second round – vignette-based interviews

The second round of interviews incorporated vignettes co-developed by the research team based on preliminary themes from the first round. Each vignette presented a realistic, context-specific scenario using the participants’ own language and reflections. Three sets of five vignettes were created and tailored to community members, researchers, or community partners. Participants read each vignette and responded to structured questions designed to elicit responses about decision-making, trust, and relationship dynamics in research partnerships. The vignette method enabled deeper reflection on sensitive or abstract issues while reducing the discomfort associated with direct questioning (27). Examples of vignettes and their intended purposes are included in Table 2.

**Table 2:** Examples of vignettes used in the second round of interviews, including their purpose and the participant groups for which they were tailored.

Vignette example (paraphrased)	Purpose	Target population
A researcher at ASU receives funding to conduct community-based participatory research but has no prior relationship with the community. To build trust, the researcher initiates phone calls with key community members and attends local events to introduce the project and the research team.	To explore strategies for initiating trust-building and relationship development in new community partnerships	Academic Researcher
A team of academic researchers receives a large grant to study how access to healthy grocery stores affects health outcomes. They must define what constitutes a “healthy grocery store” from the community’s perspective and identify ways to improve accessibility.	To examine approaches to co-defining research concepts and identifying community-relevant outcomes	Community Leader/Stakeholder
A team of academic researchers receives a large grant to study how access to healthy grocery stores affects health outcomes. They must define what constitutes a “healthy grocery store” from the community’s perspective and identify ways to improve accessibility.	To understand community members’ views on health priorities, accessibility, and relevance of academic research	Community Member
A newly funded ASU research team begins a community-based participatory research project in a community they have not previously worked with. The researchers initiate phone calls and attend community events to introduce themselves and the project, seeking to learn about community needs and priorities.	To reflect on effective ways researchers can approach unfamiliar communities and foster mutual understanding and trust	Community Member
The Our Voice Initiative empowers community members as “citizen scientists” to document local environmental factors affecting health using the Discovery Tool mobile app. Community members review their findings, prioritize issues for change, and mobilize for improvements in community health.	To explore how community-driven data collection and advocacy can shape partnerships and influence health interventions	Community Member

### 2.6 Interview logistics and documentation

All interviews were conducted via Zoom, recorded with participants’ informed consent, and transcribed using automated transcription software. Transcripts were subsequently reviewed and corrected to ensure accuracy. Supplementary data collection included a brief demographic questionnaire and field notes taken by the principal investigator (PI). These field notes captured nonverbal cues, contextual observations, and other relevant details such as setting, body language, and attire.

### 2.7 Ethical considerations and participant comfort

To address participant concerns about recording, note-taking, and confidentiality, the PI provided detailed explanations of the data collection process and associated safeguards. Participants received copies of the Institutional Review Board (IRB) protocol and the informed consent form in advance of their interview. Throughout the process, they were reminded of their rights, including the option to decline any portion of the study without consequence.

By integrating semi-structured and vignette-based interviews with contextual observations and demographic information, the study employed a comprehensive and ethically grounded approach to data collection that prioritized both methodological rigor and participant trust.

### 2.8 Rigor and trustworthiness

The rigor of this qualitative descriptive study was ensured by applying the trustworthiness criteria outlined by Guba and Lincoln (28). These criteria—*credibility, transferability, dependability, and confirmability*—provide a framework more suitable than conventional measures of reliability and validity when assessing qualitative research. *Credibility* was established through multiple strategies aimed at ensuring an authentic representation of participants’ experiences. The principal investigator (PI) built rapport with participants prior to the interviews, thoroughly reviewed the informed consent process, and addressed any concerns regarding participation. Persistent observation was maintained by recording and carefully transcribing each interview. In addition, the research team conducted debriefing sessions to review transcripts collaboratively and refine emerging insights. As Sandelowski (29) emphasizes, credibility is achieved when descriptions or interpretations are so authentic that individuals having the experience would recognize them as their own—an aim that guided our analysis and reporting. *Transferability* was supported through thick description, which included rich contextual details about participants, settings, and data collection procedures. These descriptions allow readers to evaluate whether the study’s findings are applicable to other contexts, populations, or community-academic partnership models. *Dependability* and *Confirmability* were ensured through detailed documentation and reflexive research practices. A comprehensive audit trail was maintained, recording methodological decisions and analytic procedures throughout the study (30). This decision trail enhances transparency, allowing others to trace how findings were derived and assess their consistency. The study’s analytic processes also involved coordination among investigators, collaborative code development, and consultation with an external auditor to cross-check the integrity of findings. In alignment with Sandelowski (31), the analysis prioritized a subject-oriented truth—rooted in participants’ lived experiences—rather than one imposed by the researchers. Together, these strategies reinforced the trustworthiness of the study and aligned with best practices in qualitative research aimed at centering participant voices and promoting methodological rigor.

### 2.9 Data analysis

A descriptive thematic analysis was conducted to identify, analyze, and report patterns (themes) within the qualitative data. This method is particularly effective when the goal is to provide a rich, detailed account of participant perspectives without imposing theoretical constructs or abstract interpretations (32). The analysis emphasized participants’ own words and meanings, prioritizing their lived experiences.

Data analysis began concurrently with data collection and followed an iterative, recursive process. Interview recordings were automatically transcribed using Zoom, and the transcripts were carefully reviewed and cross-checked against the original audio to ensure accuracy. The research team adopted both active and moderate participatory observer roles (33), engaging deeply with the data to develop patterns, categories, and themes using an inductive approach. Observational field notes, including notes on setting, body language, and other contextual elements, were incorporated to enrich the analysis.

**Figure 1:**
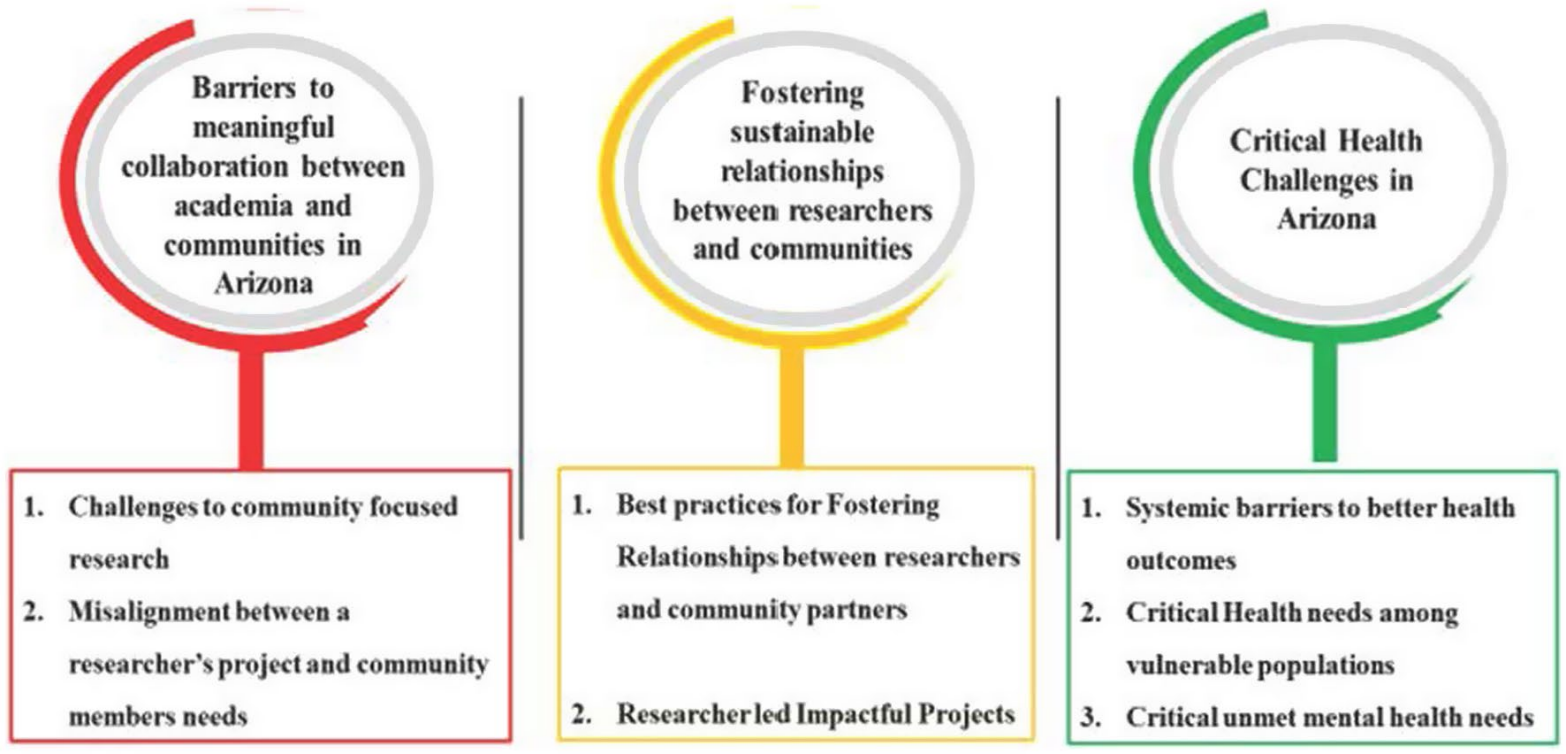
Seven emerging themes across three key focus areas.

A total of 21 interviews were manually coded, yielding 292 distinct excerpts. These excerpts were organized into seven themes across three overarching domains aligned with CBPR principles: (1) Challenges to Equitable Partnership, (2) Practices that Foster Partnership, and (3) Community-Identified Health Priorities. Coding was conducted collaboratively by multiple team members using a consensus-based process. Coders independently reviewed transcripts, developed preliminary codes, and met regularly to refine the codebook and resolve discrepancies. This approach ensured interpretive alignment and analytic rigor, while honoring the diverse perspectives of both academic and community participants (Figure 1).

The thematic analysis followed several systematic steps:

1. **Initial Review:** The research team thoroughly read each transcript, making marginal notes to document early impressions and observations.
2. **Coding:** Key excerpts from the transcripts were highlighted, color-coded, and categorized using spreadsheets. This process facilitated the identification of initial codes and emerging thematic clusters.
3. **Theme Development:** Highlighted excerpts were reviewed multiple times to refine and consolidate themes, assess internal coherence, and ensure that they accurately represented participants’ perspectives.
4. **Peer Review:** To enhance analytic rigor, the principal investigator (PI) shared the evolving themes with four team members. These discussions served to validate and refine the themes, ensuring consistency across interpretations.
5. **Iterative Refinement:** Insights from the first round of analysis directly informed the development of vignette-based questions used in the second round of interviews. This adaptive process helped explore themes in greater depth and ensured participant-centered inquiry.

This multi-step, collaborative approach ensured that the final themes were grounded in both interview and observational data, accurately reflecting the perspectives of researchers and community members alike. By prioritizing participant voice and employing rigorous, iterative methods, the analysis contributed to a deeper understanding of the complex dynamics underlying community-academic partnerships.

## 3 Results

Twenty-one participants, including academic researchers and community members, shared their perspectives on community-academic partnerships. Thematic findings were organized into three overarching domains aligned with core principles of Community-Based Participatory Research (CBPR): (1) Challenges to Equitable Partnership, (2) Practices that Foster Partnership, and (3) Community-Identified Health Priorities. Each domain reflects foundational CBPR values, including shared power, co-learning, and mutual benefit. To enhance transparency and demonstrate the analytical depth of the qualitative data, Table 3 summarizes the major themes, associated codes, and the number of coded excerpts and participants contributing to each theme. These counts reflect a high degree of thematic saturation across domains.

**Table 3:** Summary of themes, codes, and number of coded excerpts.

Domain	Theme	Associated codes (examples)	# of coded excerpts	# of participants
Challenges to equitable partnership	Structural and Institutional Barriers	Funding gaps, IRB delays, academic metrics, institutional red tape	52	17
	Misalignment of Goals and Priorities	Lack of follow-through, academic timelines, cultural disconnects	38	15
Practices that Foster Partnership	Best Practices for Engagement	Cultural humility, transparency, mutual trust, presence	60	19
	Researcher-Led Projects that Prioritize Impact	Sustainability, co-design, feedback loops, community ownership	42	16
Community-Identified Health Priorities	Systemic Barriers to Health Equity	Poverty, healthcare access, education, geographic disparities	30	13
	Leading Health Challenges	Refugee health, LGBTQ+ issues, chronic illness, disability	34	14
	Unmet Mental Health Needs	Stigma, access, provider shortage, physical manifestations	36	15

### 3.1 Domain 1: challenges to equitable partnership

CBPR alignment: Barriers to shared power, equitable involvement, and sustained participation.

#### 3.1.1 Theme one: structural and institutional barriers to engagement

Participants identified structural and institutional barriers as key impediments to building and sustaining equitable community-academic partnerships. While collaboration between academic researchers and communities is essential for addressing local health needs, systemic challenges—particularly within funding structures and institutional processes—undermine these efforts.

A major barrier noted was the limited availability of funding specifically designed for community-based research. Participants explained that while federal funding is well integrated into university systems, it often does not align with the priorities or realities of community partners. Opportunities for community-driven funding are less visible or accessible within academia, leaving researchers with few institutional supports for initiating or sustaining grassroots engagement. One researcher emphasized this point: “I’ve run into a number of hurdles, some red tape hurdles, some mechanisms within the university that have been a bit of a challenge to overcome, because it seems like the structure here is well placed for federal agency funding, not engaging with the community” (Participant 02).

Bureaucratic processes within universities also pose significant challenges, with administrative delays and rigid protocols making it difficult to establish and maintain meaningful partnerships. These hurdles are compounded by academic evaluation systems that prioritize publication output and external grant acquisition over community engagement. As one participant noted: “I think the number one priority in that sense would be that the metrics with which an academic is judged need to change” (Participant 07, Academic Researcher).

The scarcity of institutional support discourages many researchers from pursuing or sustaining community partnerships. As another participant described: “It has been a challenge actually to build partnerships between an academic institution and another institution that serves communities or with communities themselves directly” (Participant 15, Academic Researcher).

From the community perspective, this disconnect often manifests as transactional or extractive research practices. Some participants expressed concern that researchers often enter communities for data collection without long-term follow-up or impact. One community leader voiced this frustration:

“A lot of folks are hesitant to participate in research because they kind of see researchers as really opportunistic individuals who, I jokingly call research vultures” (Participant 03, Community Leader).

Trust-building, particularly with marginalized or transient populations, was highlighted as a persistent challenge. Issues of confidentiality, cultural sensitivity, and compensation were raised as critical factors influencing participation. One academic researcher explained: “Using cash, for example, is very important for my participants, because they live in a cash economy as the population is very hard, very transient. People go in and out of jail, maybe, or they change their living. They might be unsheltered or in other various circumstances that make follow-up very difficult” (Participant 11, Academic Researcher).

The COVID-19 pandemic further exacerbated these barriers, limiting opportunities for in-person engagement and straining relationships built on trust and presence. As one researcher reflected:

“It was just really difficult like getting in touch with someone… and getting them to meet with us, especially during the pandemic” (Participant 08, Academic Researcher).

Collectively, these structural and institutional barriers reveal the need for systemic change in how academic institutions support community-engaged research. Addressing funding misalignments, reforming incentive structures, and investing in long-term, relationship-centered engagement are essential to strengthening equitable partnerships.

#### 3.1.2 Theme two: misalignment of research goals and community priorities

Participants frequently described a disconnect between the goals of academic research projects and the lived realities, values, and priorities of the communities involved. This misalignment often stemmed from limited or inconsistent engagement throughout the research process, resulting in outcomes that failed to resonate with or benefit the communities under study. From the perspective of both community members and researchers, the institutional drivers of academic research—such as funding acquisition and publication pressure—were seen as contributing to this gap.

One community leader articulated this concern:

“The push still seems to be attracting funding… It does not really seem to be affecting the community in a positive light, or some of the research findings do not require, at the end of the grant, going out into the community and embedding them. Some of the findings could improve the quality of life for certain individuals, but they do not” (Participant 03, Community Leader).

Several participants reflected on how the research process itself often privileges academic advancement over practical impact:

“Researchers sometimes become addicted to research. They get addicted to securing more funding to study the same thing. They just add a few more variables that they did not consider last time” (Participant 01, Community Member).

“But it never actually gets implemented because most academics do not work in the community. They do not know how to embed the research findings in a way that actually helps” (Participant 10, Community Leader).

This lack of embeddedness and follow-through contributed to community skepticism, particularly when research was conducted without clear benefit or sustained presence. One participant described this pattern as a missed opportunity for meaningful engagement:

“They’re really looking into these community-related health issues, but you do not see them benefiting communities” (Participant 06, Community Member).

In some cases, the research itself was viewed as culturally or contextually misaligned, making it difficult to gain support or participation from community members:

“The researcher’s project did not align with the community’s values or beliefs, which made it difficult to gain their support or participation. There was a clear disconnect between the researcher and the community, and this made it difficult for the project to have a meaningful impact” (Participant 18, Community Member).

“It’s about what the academic institution or researcher wants versus what the community wants. And it seems like they are focused on publishing” (Participant 08, Academic Researcher).

The timeline and procedural demands of academic research were also noted as contributing to this misalignment. Community members, especially those facing urgent health and social challenges, felt that academic processes moved too slowly to be relevant:

“The time it takes to go through IRB, then collect all the data, analyze it, validate it, and then publish it—by that point, the communities need a faster solution” (Participant 03, Community Leader).

Together, these accounts reveal how divergent timelines, academic incentives, and limited community involvement can result in research that lacks local relevance or application. Participants emphasized the need for continuous engagement and deeper alignment between research efforts and community-defined needs—central tenets of community-based participatory research.

### 3.2 Domain 2: practices that Foster Partnership

CBPR alignment: Trust-building, co-learning, equitable involvement.

#### 3.2.1 Theme three: best practices for sustaining engagement

Participants identified several best practices for fostering meaningful, long-term community-academic partnerships. These practices—centered around empathy and cultural humility, mutual trust and transparency, fairness and empowerment, and continuous engagement—align with core CBPR principles and were consistently emphasized as essential for effective collaboration. These strategies support sustained relationships, foster co-learning, and promote mutual benefit between researchers and communities.

##### 3.2.1.1 Empathy and cultural humility

Participants described empathy and cultural humility as foundational to building trust and initiating authentic partnerships. Researchers were encouraged to enter communities with an open mind and awareness of local values, histories, and lived experiences.

“Start early on to create this collaboration… establish relationships before you do research, and then work with the community to determine what research is needed. Be mindful of historical trauma from previous research” (Participant 01, Community Member).

“The researcher has to have cultural understanding and an awareness of the environment in which community members are living” (Participant 04, Community Partner).

Some participants reflected on the social distance between academic institutions and communities, underscoring the need for researchers to be physically and socially present in community spaces.

“A lot of communities are probably intimidated by higher education, rightly or wrongly… Communities will not engage until those academics are brought into the community and see how people interact or what’s involved” (Participant 13, Community Member).

##### 3.2.1.2 Mutual trust and transparency

Mutual trust and transparency were consistently identified as prerequisites for successful engagement. Participants emphasized the importance of visible, consistent researcher presence and transparent communication about project goals and outcomes.

“It’s all about establishing trust and following up with people” (Participant 09, Community Member).

“You need to be there in person. They need to see your face. They need to trust the organization you are coming from. You need to wear a shirt that says [logo of the research institution]” (Participant 01, Community Member).

These accounts highlight that building trust is an ongoing process that requires time, accountability, and institutional credibility.

##### 3.2.1.3 Fairness and empowerment

Ensuring fairness and promoting community empowerment were viewed as key to achieving shared ownership of the research process. Participants stressed the need to involve communities in setting research priorities, shaping study design, and interpreting results.

“They know about our quarterly meetings… then we talk about their priorities. How can we help as researchers? If we were to start a research project in the future, what would be a need that they have?” (Participant 11, Academic Researcher).

“The community got to direct much more of what they are doing as researchers” (Participant 13, Community Member).

Participants also emphasized the value of creating opportunities for agency, noting that empowerment leads to more meaningful implementation:

“Engage them in the research process where they feel they have agency and control… and then show them that it’s leading toward implementation—whether that’s policy change or service implementation at a local clinic” (Participant 09, Community Member).

However, challenges with academic timelines and bureaucratic processes were acknowledged as barriers to responsiveness:

“The time it takes to go through IRB and then collect all the data, analyze it, validate it, and then publish it—by that point, the communities need a faster solution” (Participant 03, Community Leader).

##### 3.2.1.4 Continuous engagement

Participants emphasized that sustained involvement throughout the research lifecycle—not just during recruitment or data collection—was critical to fostering trust and ensuring impact.

“Community partners should have continuous engagement in all phases of research; it’s critical” (Participant 02, Academic Researcher).

“People feel that researchers collect the data and leave. There is no continuous engagement or outcome from the research” (Participant 05, Community Member).

Participants stressed the importance of showing up regularly and building relationships over time:

“Showing up, that is so critical. It’s a relationship that you are investing in. It’s not just a one-time thing. You do not just take what you need and leave; it’s an ongoing dialogue” (Participant 01, Community Member). This was especially important for engaging rural or under-resourced communities: “The best way to reach rural populations is to show up to some of the major community events” (Participant 10, Community Leader).

#### 3.2.2 Theme four: researcher-led projects that prioritize impact and sustainability

Participants emphasized that for community-academic partnerships to be effective, researcher-led projects must prioritize both **impact** and **sustainability**. Projects that address unmet community needs, incorporate community voice from the outset, and continue to provide value beyond the funding period were viewed as essential. These efforts reflect CBPR’s core principles of co-learning, mutual benefit, and long-term commitment.

##### 3.2.2.1 Centering community needs in planning and design

Community members consistently noted that impactful projects begin with early and ongoing collaboration. Rather than entering communities with predefined research agendas, participants called for researchers to spend time listening, building trust, and co-identifying issues of importance.

“Having the relationship established in the beginning, listening to each other, engaging continuously about what the issues are in the community helps to create sustainable impact” (Participant 08, Academic Researcher).

Projects grounded in local realities were perceived as more likely to generate outcomes that resonate with community members and lead to meaningful change. Participants underscored that alignment with community priorities strengthens trust and increases the likelihood of uptake.

“If researchers really want the community to care about the research, they need to involve us early. Ask us what’s important. Do not assume you know” (Participant 06, Community Member).

##### 3.2.2.2 Sharing findings and creating feedback loops

Participants also emphasized the importance of researchers returning to the community to share findings, which reinforces transparency, accountability, and shared learning.

“They do the research and then disappear. We want to know what came out of it. What did you learn? What are you going to do with it?” (Participant 03, Community Leader).

Establishing feedback loops between researchers and community members was described as an essential component of sustainability—turning research findings into community knowledge and tools for advocacy or intervention.

##### 3.2.2.3 Sustaining impact through local infrastructure

A key marker of sustainability, according to participants, was ensuring that the benefits of a research project continue after the funding ends. This requires building capacity within communities and forming partnerships with trusted local organizations.

“You have to think beyond your grant. If it ends, what’s staying behind? Are the partners equipped to continue the work?” (Participant 10, Community Leader). Participants also recommended that researchers engage across multiple layers of the community—individuals, groups, and broader networks—to ensure broader relevance and ownership.

“The researchers that do it well are the ones who meet people where they are—on the ground, at local meetings, with different groups. Not just the leaders but everyone who matters in that space” (Participant 13, Community Member).

This multi-level engagement fosters deeper trust and helps institutionalize the project’s relevance and value beyond its formal lifespan. Fostering successful community-academic collaborations is a complex yet essential task that demands intentional effort and adherence to best practices. Participants emphasized that when researcher-led projects are designed with community input and sustained through strong local partnerships, they are more likely to result in lasting, positive change. These findings highlight the importance of aligning research efforts with community-identified priorities and building projects that extend beyond the academic timeline.

##### 3.2.2.4 Designing for longevity and community ownership

Sustainability was also linked to co-ownership of the research process. Participants expressed that communities are more likely to adopt and carry forward projects when they feel a sense of agency.

“Let us own the project too. Do not just come in with your ideas and your timeline. Make us part of it from the start, so we care when it ends” (Participant 01, Community Member).

Researcher-led projects that embed sustainability from the beginning—by prioritizing relationships, co-creation, and capacity-building—were seen as more successful in delivering lasting benefits.

#### 3.2.3 Theme five: systemic barriers to health equity

Participants consistently described how systemic inequities—rooted in social, economic, and environmental determinants—undermine health and well-being across Arizona’s diverse communities. These barriers are not isolated challenges but interconnected structural issues that demand coordinated, equity-focused solutions. Interviewees highlighted a range of persistent issues including poverty, limited educational opportunities, cultural and geographic disparities, and lack of access to appropriate healthcare.

Social and structural determinants of health were seen as foundational drivers of community health outcomes. One researcher described the compounding nature of these challenges:

“Life setting interventions were not sustained due to structural and social determinants of health. A lot of health challenges are driven by community conditions—obesity, diabetes, mental health issues, and behavioral health concerns are all influenced by social factors.” (Participant 08, Academic Researcher).

Several participants emphasized the impact of limited education in both accessing and navigating healthcare systems. A community member observed:

“Most folks in my community lack the education needed to access healthcare… leading to high rates of mental health issues and depression.” (Participant 16, Community Member).

This insight reinforces the importance of addressing informational and literacy barriers alongside systemic health interventions.

Geographic and cultural diversity across the state also shapes health inequities in distinct ways. As one participant noted: “In Snowflake, Arizona, you’ll find mostly white, middle-aged to older adults involved in farming. But in Yuma, south of Tucson, you’ll see a predominance of Hispanic farmworker populations, with farming as the primary source of income.” (Participant 01, Community Member). This comparison illustrates the need for place-based, culturally responsive strategies that reflect the specific demographics and occupational realities of different communities.

Economic hardship was a recurring theme, particularly in relation to children and families in school settings. One participant shared: “Our community is diverse—our students come from a range of ethnic and socioeconomic backgrounds, with a high number qualifying for free or reduced lunch. Many of these families live below the poverty line.” (Participant 17, Community Partner).

Healthcare access—both financial and cultural—was another major concern. Participants described how cost, documentation status, and lack of culturally appropriate services often discouraged care-seeking. One interviewee summarized this challenge:

“Healthcare is expensive, and many people try to ignore health issues despite knowing that it will have long-term consequences.” (Participant 14, Community Partner).

These reflections underscore the deeply rooted and intersecting nature of systemic barriers to health equity. To reduce disparities, participants emphasized the need for tailored, place-specific approaches that account for socioeconomic conditions, education levels, cultural context, and local infrastructure. Without addressing these foundational issues, interventions risk being short-term and ineffective.

#### 3.2.4 Theme six: leading health challenges in marginalized communities

Grounding research in the voices and lived experiences of community members is fundamental to the Community-Based Participatory Research (CBPR) model. This approach revealed the specific health challenges confronting communities in Arizona, shaped by distinct social, cultural, and structural factors. Such context-driven insights are essential to informing research and developing interventions that are both effective and equitable.

Underrepresented communities face distinct barriers to healthcare access that compound their health challenges. For example, undocumented individuals often avoid seeking care due to fears related to immigration status. One community member explained, “Immigration status forces asylum seekers to rely heavily on emergency rooms, as they lack access to preventative care” (Participant 09, Community Member). Newcomers, including immigrants and refugees, frequently experience mental health challenges such as stress, anxiety, depression, PTSD, and anemia, all requiring culturally sensitive approaches. The LGBT community also faces specific health concerns—depression, anxiety, chronic diseases, and metabolic disorders—that demand tailored support. Reflecting on the broader healthcare system, a participant expressed frustration: “Healthcare delivery here is fragmented and complex, and even educated individuals often struggle to navigate the system” (Participant 13, Community Member).

The reliance on emergency services instead of preventative care remains a persistent challenge across many marginalized groups. For instance, people with disabilities face unique health needs that are often overlooked, prompting calls for “more education on how to work with people with disabilities and expand our service offerings to meet their needs” (Participant 18, Community Partner).

By centering community voices and lived experiences, CBPR encourages researchers to engage deeply with these contextual realities to co-create interventions that are culturally relevant, accessible, and sustainable. This approach fosters trust and ensures that health solutions truly address the priorities and challenges identified by marginalized communities themselves.

#### 3.2.5 Theme seven: unmet mental health needs

Including community members in this study revealed that unmet mental health needs are among the most urgent and widespread health concerns across Arizona’s underserved populations. In line with the Community-Based Participatory Research (CBPR) model, which emphasizes grounding research in the lived experiences of communities, participants identified mental health as a pressing yet often overlooked priority.

Participants described how stigma surrounding mental health—especially anxiety, depression, and stress—prevents individuals from seeking care. These conditions often manifest through physical symptoms, further complicating diagnosis and treatment. As one participant explained, “There are no good systems providing the mental health care needed. Stress manifests in ulcers, stomach problems, panic attacks, and even seizures, sometimes to the point of losing consciousness” (Participant 19, Community Partner).

Access to appropriate care is further limited by a severe shortage of clinical therapists and long waitlists. “The long wait times and a shortage of therapists make it incredibly difficult to get the help people need. Stigma also makes it harder for individuals to seek assistance” (Participant 12, Community Member).

Structural challenges—such as high staff turnover and inadequate cultural competency training—also emerged as key barriers to effective mental health care. “High staff turnover and the lack of cultural sensitivity in training contribute to inadequate care. People often do not receive the services they need, or it’s incredibly difficult for them to access care at all” (Participant 19, Community Partner).

In sum, by centering the voices of those directly affected, this study surfaces mental health as a critical area for action and accountability. CBPR provides a framework for co-developing solutions that are culturally responsive, sustainable, and rooted in the realities of the communities most impacted.

### 3.3 Diverging perspectives

While academic and community participants shared a strong commitment to trust, equity, and community engagement, their perspectives diverged notably in how they identified barriers to equitable partnerships and defined success. These differences underscore the value of CBPR’s emphasis on co-learning and mutual respect, which requires recognizing and reconciling divergent priorities and lived realities.

Academic researchers frequently pointed to structural and institutional obstacles as key barriers. Challenges such as limited funding for community-engaged work, delays in Institutional Review Board (IRB) approval, and academic performance metrics that undervalue partnership-building were central concerns. As one researcher explained, “The metrics with which an academic is judged need to change” (Participant 07), pointing to the systemic pressures that limit deeper engagement.

In contrast, community members and leaders expressed frustration with what they viewed as transactional research relationships and a lack of tangible follow-through. A recurring theme was the perception that researchers “collect data and leave” without delivering sustained benefits to the community (Participant 03, Community Leader). This critique highlights a relational gap—one rooted not in procedural limitations but in a deeper mistrust shaped by past experiences and unmet expectations.

Timelines also revealed key points of tension. Academic researchers described long delays associated with grant cycles and publication processes, whereas community members stressed the need for more responsive, real-time interventions. “By the time data is collected, analyzed, and published, our communities still have not seen change,” explained one community leader, echoing a broader concern raised by multiple participants about academic timelines not aligning with community urgency (Participant 03, Community Leader).

Despite these differing perspectives, both groups underscored the importance of cultural humility, transparent communication, and consistent engagement. These shared values offer common ground for addressing the mismatches in expectations and approaches.

In conclusion, these diverging perspectives illustrate the importance of sustained dialogue and reflexivity in community-academic partnerships. A CBPR approach—centered on shared decision-making, mutual accountability, and respect for both scientific and lived knowledge—can help bridge these gaps, ensuring that research is both rigorous and responsive to community priorities and needs.

## 4 Discussion

The field of community-engaged research continues to evolve, guided by principles rooted in Community-Based Participatory Research (CBPR) and related frameworks. While significant progress has been made, a persistent gap remains in understanding how community engagement shapes both the research process and its real-world impacts on health outcomes— particularly for underserved and structurally marginalized populations. This study helps address that gap by offering new insights into the strategies, tensions, and systemic barriers that influence long-term, trust-based academic-community partnerships in the context of persistent health disparities in Arizona.

Findings from this study reinforce the complexities of building sustainable, equitable collaborations. Participants voiced the importance of trust, cultural responsiveness, and long-term commitment, while also exposing systemic barriers—such as institutional constraints, cultural disconnects, resource limitations, and misaligned research priorities—that continue to hinder effective partnership and equitable outcomes. These insights provide practical guidance for researchers, practitioners, and policymakers committed to co-creating meaningful and actionable health solutions with communities. One key contribution of this study is its affirmation of best practices for academic-community partnerships, particularly around shared power, transparency, co-leadership, and bidirectional learning. These principles align with and extend existing scholarship that calls for a shift from conducting research on communities to conducting research with communities (23, 34). Such partnerships are better positioned to foster relevance, ownership, and sustainability—outcomes that are difficult to achieve through extractive or top-down research models. Barriers to engagement—including institutional limitations, trust erosion, and insufficient infrastructure—remain persistent and were strongly voiced in this study. These challenges mirror those reported in other community-engaged research efforts, where systemic inequities often constrain participation and mutual benefit. Prior research supports strategies such as sustained capacity building, investment in community leadership, and structural accountability mechanisms to navigate and mitigate these barriers (13, 14). At the same time, this study uncovers persistent gaps in engagement—particularly the frequent misalignment between academic research agendas and community needs. Several academic researcher participants shared that institutional bureaucracy, rigid funding mechanisms, and academic promotion metrics often disincentivize community engagement, while community members expressed frustration with transactional research practices and limited follow through. This reinforces the call for more inclusive planning processes, continuous dialogue, and the use of culturally and contextually responsive, community-centered approaches throughout all phases of research (5, 35), and institutional reforms that value community engagement as central to scholarly success. One notable contribution is the emphasis on hybrid models of researcher-led, community-guided projects—highlighting the potential for academic leadership that is grounded in community priorities and supported through inclusive, relational engagement. Rather than relying solely on traditional top-down models, these approaches elevate the role of community expertise in shaping interventions, consistent with emerging literature on co-creation as a driver of sustained community change (3, 4). Importantly, the findings from Focus Area C highlight community-identified health challenges specific to Arizona’s underrepresented populations. Participants described structural barriers such as limited mental health services, long wait times, high provider turnover, and a lack of culturally and linguistically appropriate care. Community members shared how immigration status, poverty, and fear of deportation forced many to rely on emergency rooms for care rather than accessing preventive services. Others voiced concern over fragmented healthcare systems, the stigma surrounding mental health, and the absence of disability-inclusive practices. These findings contribute to the growing evidence base linking health outcomes to broader social determinants and underscore the need for multisectoral, context-specific responses (1). Addressing health inequities will require moving beyond a narrow focus on healthcare access alone to tackle the structural roots of inequity. These lived experiences point to an urgent need for integrated, community-informed health strategies that address real-world health challenges and prioritize trust, continuity, and relevance to community needs.

This study also contributes methodologically by incorporating vignettes as a participatory tool that facilitated deeper reflection and helped surface power dynamics and hidden tensions in a non-threatening, context-sensitive manner. Participants responded with rich narratives and emotional insights, offering a more layered understanding of barriers to and facilitators of engagement. This innovative application of vignettes adds to the qualitative toolkit for health equity and implementation research.

In summary, the findings from this study provide a grounded, empirically rich contribution to the literature on community-academic partnerships. They highlight both enduring barriers and promising practices while emphasizing the need for structural change, community co-leadership, trust-based, community-responsive approaches to engagement. These lessons are especially relevant in under-resourced settings like Arizona and are transferable to other regions seeking to dismantle inequities through community-rooted research models.

### 4.1 Contribution to existing literature

This study contributes to the existing literature on community-academic partnerships and health equity in several important ways. First, it offers a nuanced analysis of how systemic and relational dynamics—such as institutional constraints, power imbalances, and community trust—interact to shape the quality and sustainability of academic-community collaborations. By centering perspectives from Arizona’s diverse and historically marginalized communities, it provides new empirical evidence from a regional context that remains underrepresented in community engagement research (36, 37). Second, the study highlights how misalignments between institutional metrics for academic promotion and community-defined priorities generate persistent tensions that hinder authentic, equity-oriented research. These findings echo and extend prior scholarship emphasizing the need to reform institutional reward structures to support meaningful engagement (6). Third, the incorporation of vignettes as a participatory qualitative method proved particularly effective in eliciting rich, situated reflections on trust, accountability, and partnership dynamics—offering a methodological contribution to the field. These findings are consistent with prior research that identifies trust, transparency, role clarity, and institutional alignment as foundational to effective community-academic partnerships (7, 9). Our study extends this work by offering a region-specific perspective that underscores how local sociopolitical contexts and historical inequities uniquely shape these dynamics in underrepresented areas like Arizona. Finally, this study deepens current understandings of how social determinants of health intersects with local political, economic, and cultural realities, yielding actionable insights for developing grounded, context-specific strategies in community-engaged research and public health equity practice.

## 5 Limitations

Several limitations of this study should be acknowledged. First, the research was conducted in a single regional setting—Arizona—which, while demographically diverse and active in community-engaged scholarship, may limit the broader applicability of the findings. Geographic, cultural, and institutional differences across regions may influence the nature and dynamics of community-academic partnerships in ways not captured here.

Second, the academic researcher sample was composed primarily of individuals already engaged in or supportive of community-academic partnerships. This may introduce a selection bias, as researchers with limited experience, critical views, or institutional resistance toward community engagement may be underrepresented. Consequently, the findings may reflect more favorable or optimistic perspectives regarding the feasibility and value of these partnerships.

Third, although the study intentionally included a wide range of community voices—including Latinx, Native American, LGBTQ+, immigrant, refugee, and rural populations—some perspectives may remain underrepresented. Non-English-speaking individuals, those with limited digital literacy or access, and individuals with high levels of institutional mistrust may not have been adequately reached, potentially limiting the depth and inclusivity of the findings.

Finally, while the use of vignettes enriched the data by prompting reflection on real-world scenarios and power dynamics, the findings remain interpretive and exploratory in nature. The qualitative design does not allow for causal conclusions or broad generalizations. Future research using longitudinal or mixed-methods approaches would help to assess how community-academic partnerships develop over time and to evaluate their sustained impact on community health outcomes and equity.

### 5.1 Future research directions

Building on the current findings, future studies could explore how specific strategies—such as embedding co-leadership structures, institutional policy reforms, or participatory methods like vignettes—affect long-term sustainability and equity in community-academic partnerships. Research could also examine the implementation of engagement practices across different institutional and community contexts, and test models that align institutional incentives with community-defined priorities. Comparative or longitudinal studies may offer further insight into how partnerships evolve over time and which factors most effectively promote mutual benefit and impact.

## 6 Conclusion

This study offers critical insights into the structural and relational dynamics that shape community-academic partnerships in the context of health equity. Grounded in the voices of both community members and academic researchers across Arizona, the findings expose persistent barriers—such as institutional bureaucracy, misaligned research agendas, mistrust, and inequitable power structures— that continue to hinder authentic, sustained collaboration. Through 21 in-depth interviews, we identified challenges and facilitators to equitable partnerships, highlighting practices that promote trust, co-leadership, and alignment with community-defined priorities.

While rooted in the Arizona context, the lessons identified here are transferable to other regions where health disparities intersect with broader patterns of social, cultural, and political marginalization. By centering community voices, embracing co-leadership, and aligning research with community-defined priorities, this study contributes to a growing movement to reimagine how health research is conceptualized, funded, and conducted.

Ultimately, fostering successful community-academic collaborations is a complex but essential endeavor that demands commitment at all levels—from individual researchers to institutional leaders and funders. By embedding equity, relationship-building, and sustainability at the core of research design and implementation, academic institutions can help ensure that their work not only advances scholarly knowledge but also delivers meaningful, lasting benefits to the communities they aim to serve.

## Data Availability

The original contributions presented in the study are included in the article/supplementary material, further inquiries can be directed to the corresponding author.
